# Repurposing major metabolites of lamiaceae family as potential inhibitors of *α*-synuclein aggregation to alleviate neurodegenerative diseases: an *in silico* approach

**DOI:** 10.3389/fphar.2025.1519145

**Published:** 2025-04-16

**Authors:** Soham Bhattacharya, Neha Gupta, Adrish Dutta, Pijush Kanti Khanra, Ritesh Dutta, Jana Žiarovská, Nikolay T. Tzvetkov, Lucie Severová, Lenka Kopecká, Luigi Milella, Eloy Fernández-Cusimamani

**Affiliations:** ^1^ Department of Agroecology and Crop Production, Faculty of Agrobiology, Food and Natural Resources, Czech University of Life Sciences Prague, Prague, Czechia; ^2^ Department of Crop Sciences and Agroforestry, Faculty of Tropical AgriSciences, Czech University of Life Sciences Prague, Prague, Czechia; ^3^ Department of Biosciences and Bioengineering, Indian Institute of Technology Guwahati, Guwahati, Assam, India; ^4^ Environmental Biotechnology and Genomics Division, CSIR-National Environmental Engineering Research Institute (CSIR-NEERI), Nagpur, India; ^5^ Institute of Plant and Environmental Sciences, Faculty of Agrobiology and Food Resources, Slovak University of Agriculture in Nitra, Nitra, Slovakia; ^6^ Department of Biochemical Pharmacology and Drug Design, Institute of Molecular Biology “Roumen Tsanev”, Bulgarian Academy of Sciences (BAS), Sofia, Bulgaria; ^7^ Department of Economic Theories, Faculty of Economics and Management, Czech University of Life Sciences Prague, Prague, Czechia; ^8^ Department of Science, University of Basilicata, Potenza, Italy

**Keywords:** functional network analysis, molecular docking, MD simulation, neuroprotective agent, pathway enrichment analysis, phytochemical

## Abstract

Neurodegenerative disorders (NDs) are typically characterized by progressive loss of neuronal function and the deposition of misfolded proteins in the brain and peripheral organs. They are molecularly classified based on the specific proteins involved, underscoring the critical role of protein-processing systems in their pathogenesis. Alpha-synuclein (α-syn) is a neural protein that is crucial in initiating and progressing various NDs by directly or indirectly regulating other ND-associated proteins. Therefore, reducing the α-syn aggregation can be an excellent option for combating ND initiation and progression. This study presents an *in silico* phytochemical-based approach for discovering novel neuroprotective agents from bioactive compounds of the Lamiaceae family, highlighting the potential of computational methods such as functional networking, pathway enrichment analysis, molecular docking, and simulation in therapeutic discovery. Functional network and enrichment pathway analysis established the direct or indirect involvement of α-syn in various NDs. Furthermore, molecular docking interaction and simulation studies were conducted to screen 85 major bioactive compounds of the Lamiaceae family against the α-syn aggregation. The results showed that five compounds (α-copaene, γ-eudesmol, carnosol, cedryl acetate, and spathulenol) had a high binding affinity towards α-syn with potential inhibitory activity towards its aggregation. MD simulations validated the stability of the molecular interactions determined by molecular docking. In addition, *in silico* pharmacokinetic analysis underscores their potential as promising drug candidates, demonstrating excellent blood-brain barrier (BBB) permeability, bioactivity, and reduced toxicity. In summary, this study identifies the most suitable compounds for targeting the α-syn aggregation and recommends these compounds as potential therapeutic agents against various NDs, pending further *in vitro* and *in vivo* validation.

## 1 Introduction

Neurodegenerative disorders (NDs), incurable and debilitating diseases of the central and peripheral nervous system (CNS and PNS), are the worldwide leading cause of death and genesis of millions as per disability-adjusted life years (DALYs). NDs are associated with a range of clinical manifestations, including depression, dementia, bradykinesia, and ataxia (Williams-Gray and Worth, 2016). The report on the Global burden of disease (GBD) suggested the number of people affected globally by several NDs such as Alzheimer’s disease (AD), Parkinson’s disease (PD), Huntington’s disease (HD), epilepsy, multiple sclerosis, and others, will rise from 6.2 million to 13 million by 2040 (Feigin et al., 2017). According to the World Health Organisation (WHO), NDs are expected to overtake cancer by 2040 to become the second leading cause of mortality worldwide with the highest prevalence rates and pose a significant financial and health burden globally (Chopade et al., 2023; Dua and Neurology, 2004).

There is neither cure nor prevention available for ND patients; however, NDs can be treated with a variety of synthetic medications such as monoamine oxidase inhibitors, dopamine agonists, acetylcholinesterase inhibitors, adenosine receptor antagonists, L-DOPA replacement therapy, and others (Ellis and Fell, 2017). Considering the epidemiological predictions of NDs and their shortcomings, investigations into natural alternatives with effective and potent preventive actions against acute neurological deterioration are necessary while being well tolerated and free from adverse side effects to patients (Sharifi-Rad et al., 2022). The oxidative stress is a major hallmark of NDs resulting from dysregulated reactive oxygen species (ROS) production. Imbalances in cellular antioxidant defences, coupled with mitochondrial dysfunction, lipid peroxidation, neuroinflammation, and altered dopamine metabolism, contribute to disease pathology. Research focuses on identifying molecules that can modulate ROS pathways and exploring natural polyphenolic phytochemicals as potential therapeutic agents to overcome current treatment limitations (Abd Rashed et al., 2021). Aromatic plants produce essential oils with antiviral, antibacterial, antifungal, and neuroprotective properties. Many studies reported that phytochemicals play a crucial role in maintaining the brain’s chemical balance by modulating the receptor function of specific inhibitory neurotransmitters (Guo et al., 2015; Kolouri et al., 2016; Yadav, 2021). The Lamiaceae family, encompassing over 7,000 species and 236 medicinally significant genera, represents a vast reservoir of bioactive secondary metabolites, including alkaloids, terpenoids, and polyphenols, underpinning its pharmacological significance (Wink, 2020). Among its members, genus *Ajuga* are particularly noteworthy for their diverse phytochemical profile, comprising iridoids (e.g., harpagide), neo-clerodane diterpenes, and phytoecdysteroids, which contribute to their antimicrobial, anti-inflammatory, and neuroprotective effects (Mimaki et al., 2021). Furthermore, Lamiaceae-derived polyphenols, such as rosmarinic and salvianolic acids, demonstrate acetylcholinesterase inhibition, highlighting their therapeutic relevance in NDs, particularly in AD, while also exhibiting potential in metabolic regulation, including glucose homeostasis in diabetes (Zengin et al., 2018). Collectively, these bioactive constituents position Lamiaceae species as promising candidates for the development of novel interventions in NDs and metabolic disorders (Gupta et al., 2024; Hernandez-Leon et al., 2021). For instance, extracts from *Salvia Rosmarinus* Spenn., *Salvia officinalis* L., *Otostegia persica* (Burm.) Boiss., *Ocimum menthiifolium* Hochst. ex Benth., and *Nepeta menthoides* Boiss. & Buhse have been found to exhibit antidepressant and anxiety-reducing properties (Firoozabadi et al., 2015; Kolouri et al., 2016; Mank-Halati et al., 2024; Nematolahi et al., 2018; Sadeghi et al., 2014; Zahran et al., 2021). Proteins like amyloid β-, τ-protein, α-synuclein, and TDP-43 are the most frequently aggregated (misfolded) proteins culpable for NDs. Also, over the years, studies suggested that α-synuclein (α-syn), which is a heat-stable, soluble, and abundant presynaptic brain protein with 140 amino acids encoded by synuclein or SNCA gene, plays a salient role in neurotransmitter release and vesicular trafficking. However, the formation of insoluble forms of α-syn and its inclusion in Lewy bodies is associated with various NDs. It is also directly or indirectly interconnected with various proteins associated with NDs like Parkinson’s disease (PD) and other synucleinopathies (Horne et al., 2024). It disrupts cellular membranes, interferes with protein degradation pathways like the ubiquitin-proteasome system and autophagy, and activates inflammatory processes (Goedert, 2001). This leads to cellular homeostasis disruption and neuronal death, affecting synaptic function. Additionally, secreted *α*-syn can harm neighboring cells by promoting aggregation, potentially contributing to disease propagation (Shim et al., 2022; Stefanis, 2012). It can also upregulate or downregulate their expression to facilitate different NDs initiation and progression (Davidson et al., 1998; Ghiglieri et al., 2018; Uversky, 2017). Therefore, it can be a potential targeted biomarker for developing precision therapies against NDs (Brundin et al., 2017).

Fortunately, various preclinical reports described phytochemicals belonging to the Lamiaceae family like rosmarinic acid, curcumin, morin, *etc.*, have been reported to specifically target α-syn, which efficiently scavenges oxygen free radicals via different pathways (Hui et al., 2018; Meena et al., 2021), thereby, protecting cells from oxidative damage, confirming the neurogenic potential which prevents loss of neuronal processes (Naoi et al., 2017). Phytochemical-based drug discovery leveraging molecular docking and ADMET analysis presents a robust and cost-effective strategy for identifying novel therapeutic agents. This method facilitates high-throughput screening of natural compounds, detailed insights into molecular interactions, and elucidation of unknown molecular structures, including enzymes and their potential ligands. In silico methods for optimizing the structure of biotherapeutic agents have been reported in several studies, demonstrating the potential of docking and ADMET analysis in novel therapeutic development (Asati et al., 2020; Saha et al., 2023).

**FIGURE 1:**
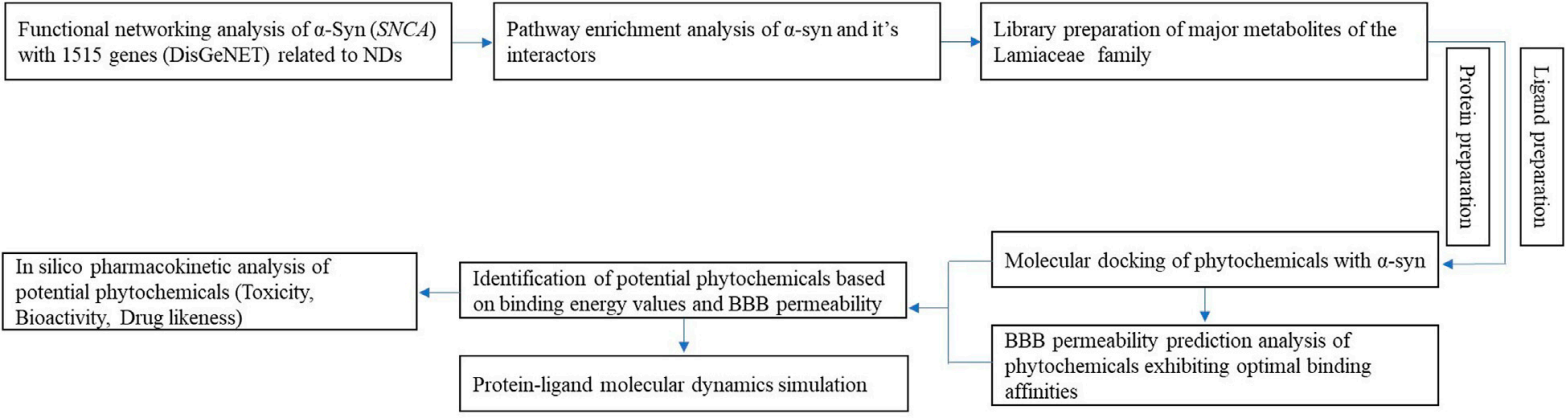
Schematic diagram of the workflow employed in this study.

Numerous studies have investigated the clinical relationships between proteins implicated in neurodegenerative diseases and phytochemicals (Guo et al., 2015; Lopez-Lopez et al., 2004). However, a unified target linking all neurodegenerative diseases and elucidating their molecular interactions with phytochemical-based bioactive compounds has not yet been identified. Comprehensive research substantiating the association between phytochemicals and neurodegenerative diseases with solid scientific validation is needed to fill this knowledge gap. Therefore, this study aims to elucidate the role of α-synuclein (α-syn) in various neurodegenerative disease pathways, highlighting its potential as a drug development target. Furthermore, it investigates the therapeutic potential of bioactive compounds found in the essential oils of Lamiaceae plants against α-syn aggregation and neurodegenerative disease progression. The research encompasses functional network analysis, pathway enrichment analysis of α-syn, molecular interaction studies with these bioactive compounds, and pharmacokinetic evaluations to assess their viability as promising lead candidates in drug design (Figure 1).

## 2 Materials and methods

### 2.1 Construction of component functional network

The functional and physical interconnections between α-syn and other proteins involved in neurodegenerative diseases were confirmed using the STRING version 9.1 database (https://string-db.org) (Franceschini et al., 2013), which identifies physical and functional associations between proteins by analyzing co-expressed genes, literature, high-throughput experimental data, and database mining. The neurodegenerative disease-related genes were identified using DisGeNET (https://www.disgenet.org/, accessed on 12 June 2024). For this study, α-syn was queried in the STRING database with *Homo sapiens* selected as the organism. An interaction score threshold of 0.7 (indicating high confidence) was set. Additionally, cluster analysis was conducted using the K-means clustering algorithm, specifying eight clusters.

### 2.2 Pathway enrichment analysis

The involvement of α-syn interactors in various biological pathways was analyzed using the Enrichr database (https://maayanlab.cloud/Enrichr/enrich#). Enrichr enables users to investigate the roles of query proteins in pathways, ontology, diseases/drugs, and expression across different cell lines. The list of primary interactors of α-syn was submitted as a query to the Enrichr server. Their roles in different pathways were analyzed using the KEGG 2021 Human database, which includes manually curated data on human cell signaling and metabolic pathways (2021 version). A bar graph depicting the top 10 pathways, ranked by p-value, was downloaded from Enrichr. Additionally, a CSV file containing the table format of all pathways associated with α-syn and its interactors was retrieved.

### 2.3 Target protein and ligand preparation

The crystal structure of *α*-syn (3Q26) implicated in neurodegenerative diseases was obtained from the RCSB PDB (accessed on 12 March 2024) and prepared for docking studies by removing unnecessary chains and residues using UCSF Chimera 1.16. For ligand preparation, 3D SDF structures of 85 major phytochemical compounds belonging to the Lamiaceae family were identified according to previous studies (Ebadollahi et al., 2020; Ramos da Silva et al., 2021) and downloaded from PubChem (accessed on 12 March 2024). These compounds were imported into Avogadro 1.2.0 and subjected to energy minimization using the MMFF94 force field with the steepest descent algorithm. The minimized structures were then converted to PDBQT format for docking.

### 2.4 Molecular docking and binding analysis

AutoDock Tools 1.5.7 (Scripps Research Institute, La Jolla, CA, United States) was utilized to predict protein-ligand interactions. Water molecules were removed, and polar hydrogens and Kollman charges were added to the protein structures. Both protein and ligand files were converted to PDBQT format. Active sites were determined using blind-docking and enclosed within a 3D affinity grid centred on the active site residues. Docking was executed via command prompt following the method described previously (Gupta et al., 2023). Binding energies were recorded, and initial visualizations were performed with BIOVIA Discovery Studio Visualizer (BIOVIA, San Diego, CA, United States). The follow-up detailed analysis of amino acid and ligand interaction was also performed using the same. Phytochemical compounds from the Lamiaceae family exhibiting optimal binding affinities to α-synuclein were selected for blood-brain barrier (BBB) permeation analysis. The top five compounds demonstrating both high BBB permeability and superior binding affinity were then chosen for further molecular dynamics simulations and drug-likeness analysis. Additionally, to cross-verify the binding affinities of the ligands with proteins, docking was conducted using the CB-Dock2 online server (https://cadd.labshare.cn/cb-dock2/index.php, accessed on 12 March 2024).

### 2.5 Drug likeness, bioactivity, and toxicity prediction analysis

Drug-likeness, bioactivity analysis, and toxicity prediction are crucial for identifying novel drug candidates. Parameters for Lipinski’s rule of five including molecular weight (MW), hydrogen bond acceptors and donors (HBD/HBA), octanol/water partition coefficient (LogP), topological polar surface area (TPSA), bioavailability, and BBB permeability were assessed using SwissADME (http://www.swissadme.ch/, accessed on 14 March 2024). Additionally, the BBB scores of all compounds were confirmed using the Molsoft web server (https://www.molsoft.com/mprop/, accessed on 14 March 2024). The bioactivity scores of the ligands were obtained from the Molinspiration web server (https://www.molinspiration.com/cgi/properties, accessed on 14 March 2024). Candidate molecules were predicted using *in silico* methods via the pkCSM online tool (https://biosig.lab.uq.edu.au/pkcsm/prediction, accessed on 15 March 2024), evaluating parameters such as AMES toxicity, maximum tolerated dose (human), hERG I and hERG II inhibitory effects, oral rat acute toxicity, hepatotoxicity, skin sensitization, and fathead minnow toxicity.

### 2.6 Molecular dynamics and simulation

The five best protein-ligand complexes from the molecular docking study were selected for MD simulation based on the binding energy and optimal docked pose. The macromolecular structure of α-synuclein (3Q26) comprised multiple subunits, which were segregated, and their additional homodomains were removed using Discovery Studio Visualizer v20.1.0.19195 (https://www.3ds.com/products/biovia/discovery-studio). Adjacent heteroatoms were also removed similarly. The modified structures were then further refined to become compatible with *in silico* virtual screening and molecular dynamics simulation (MDS). A comparison map of the dynamic characteristics of our target proteins and their protein-ligand complexes was generated using GROMACS 2020.4.1, following the methodology described before (Bhattacharya et al., 2024a; Bhattacharya et al., 2024b; Kandasamy et al., 2022). The Protein and protein-ligand complexes were extracted as GROMACS files for 200 ns (nanoseconds) in steps of 2 femtoseconds (fs) from CHRMM-GUI files.

### 2.7 MD trajectory and binding free energy analysis

After the successful completion of MD simulation of specific protein-ligand interaction complexes, the following analyses were performed: root mean square deviation (RMSD), solvent accessible surface area (SASA), radius of gyration (R_g_), hydrogen bond (HB) and root mean square fluctuations (RMSF). These analyses were performed during the entire 200 ns MD trajectory at intervals of 20 ns. Finally, using Qtgrace (https://qtgrace.sourceforge.io/), all the graphs (HB, R_g_, SASA, RMSD, and RMSF) were plotted together.

The recalculating binding free energy of protein-ligand interactions was further assessed using MD simulation-specific binding free energy analysis using the MM-PBSA (Molecular Mechanics Poisson-Boltzmann Surface Area) method. The following equation was used by the GROMACS function “g_mmpbsa” to collect MD trajectory data of protein-ligand interaction complexes at 5-ns intervals.

$$\Delta E_{\text{MM}}+\Delta G_{\text{PBSA}}‐T\Delta S_{\text{MM}}=\Delta G_{\text{BA}}$$

Where ΔG_BA_ indicates average free energy, ΔE_MM_ indicates average molecular mechanics energy, ΔG_PBSA_ indicates solvation energy, and TΔS_MM_ denotes solute configuration entropy respectively (Kandasamy et al., 2022).

## 3 Results

### 3.1 Functional networking and pathway enrichment analysis

**FIGURE 2:**
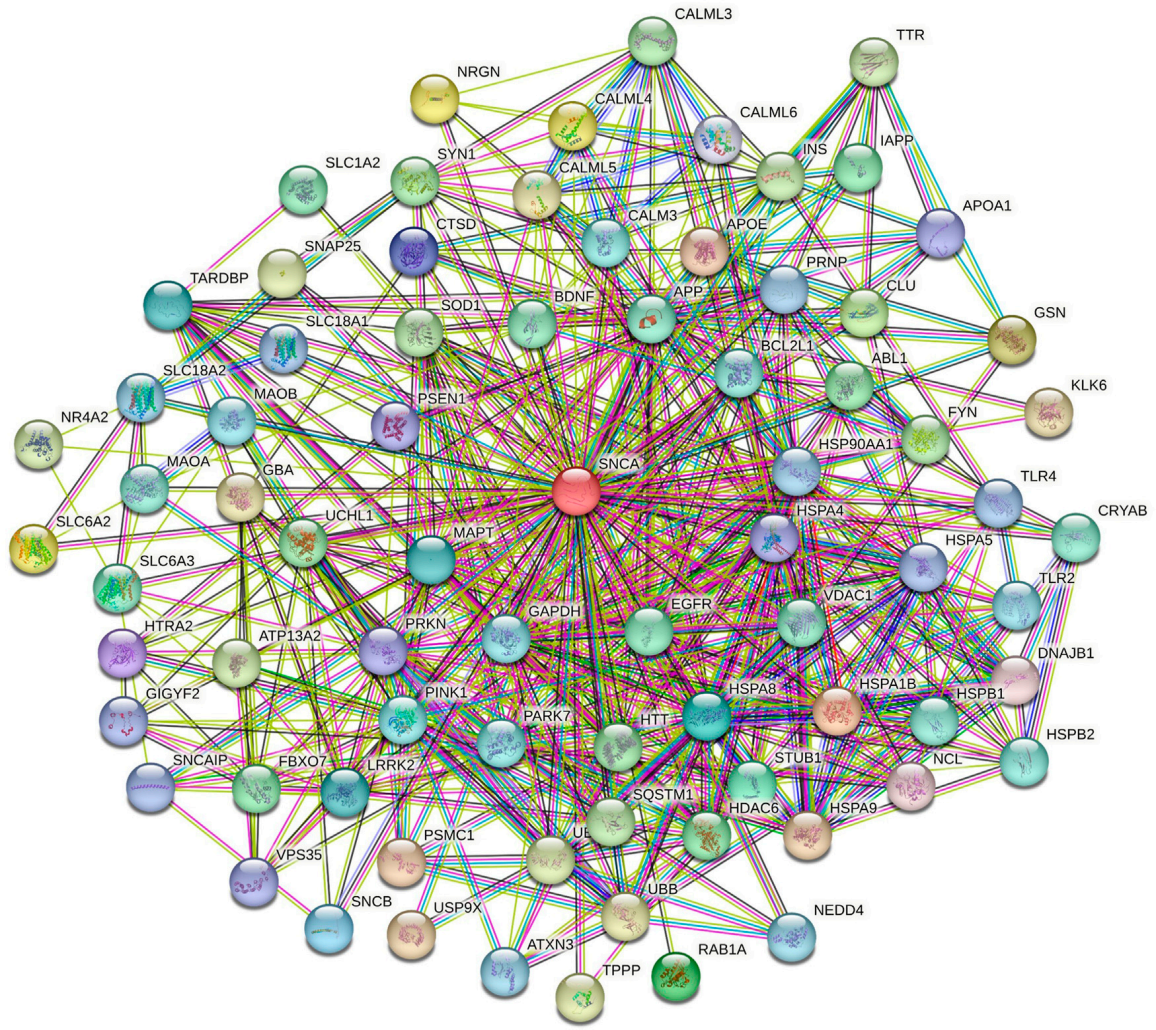
Functional network of α-syn visualized using the STRING database.

**FIGURE 3:**
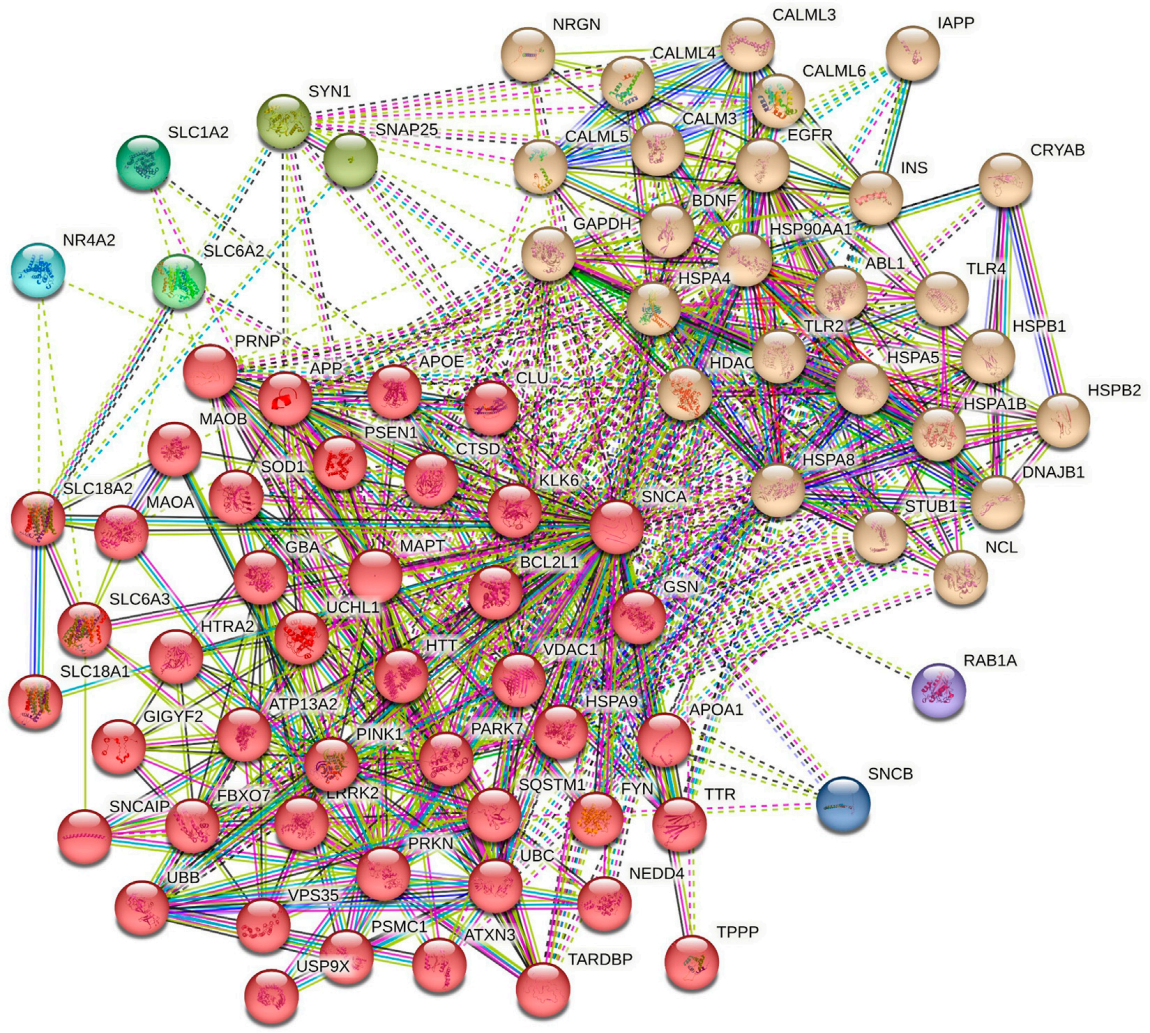
Clusters of *α*-syn primary interactors by K-means clustering.

The role of α-syn in various pathways of neurodegenerative diseases was elucidated through functional network analysis. 1,515 genes related to multiple neurodegenerative diseases were used in this study. The interconnection between α-syn (also its encoding gene SNCA) and its 76 interactor proteins are displayed in Figure 2 and Supplementary Table S1. Further, the functional network of α-syn was categorised into distinct 8 clusters to simplify the analyses of their connection in different pathways. The cluster illustrates numerous associations of pathway-related genes with α-syn (Figure 3).

Cluster 1 comprised 44 proteins, including those involved in the Ubiquitin-Proteasome System (UPS), such as F-box protein 7 (FBXO7), Neural precursor cell expressed developmentally downregulated protein 4 (NEDD4), Parkinsonism-associated deglycase (PARK7), PTEN-induced putative kinase 1 (PINK1), Parkin RBR E3 ubiquitin-protein ligase (PRKN), Sequestosome 1 (SQSTM1), Ubiquitin B (UBB), Ubiquitin C (UBC), Ubiquitin C-terminal hydrolase L1 (UCHL1), and Ubiquitin specific peptidase 9, X-linked (USP9X) (Celebi et al., 2020; Han et al., 2023; Huang et al., 2020; Ristic et al., 2014). Additionally, proteins such as Amyloid beta precursor protein (APP), Huntingtin (HTT), Microtubule-associated protein tau (MAPT), Prion protein (PRNP), TAR DNA binding protein (TARDBP), and Transthyretin (TTR) are critical in neurodegeneration and protein aggregation (Gandhi et al., 2019; Goedert, 2015; Wang et al., 2024). Proteins vital for synaptic functions and neurotransmission include Vesicular monoamine transporter 1 (SLC18A1), Vesicular monoamine transporter 2 (SLC18A2), Dopamine transporter (SLC6A3), Synuclein alpha interacting protein (SNCAIP), and Voltage-dependent anion channel 1 (VDAC1) (Courtin et al., 2006; Ning et al., 2023; Shoval et al., 2014). The proteins BCL2 like 1 (BCL2L1), Clusterin (CLU), Monoamine oxidase A (MAO-A), Monoamine oxidase B (MAO-B), and Superoxide dismutase 1 (SOD1) are involved in cell survival and apoptosis (Dabrowska et al., 2011; Suthar and Lee, 2023). For protein folding and stress response, Cathepsin D (CTSD), Gelsolin (GSN), HtrA serine peptidase 2 (HTRA2), and Heat1 shock protein family A member 9 (HSPA9) are key players. In contrast, Leucine-rich repeat kinase 2 (LRRK2) and Fyn proto-oncogene, Src family tyrosine kinase (FYN) are significant for kinase activity (Cai et al., 2022; Garrido et al., 2022; Jin and Youle, 2013). Essential proteins regulating transcriptional and translational processes include the Proteasome 26S subunit, ATPase1 (PSMC1), and GRB10 interacting GYF protein 2 (GIGYF2) (Sathitloetsakun and Heiman, 2024; Yang et al., 2021). Lastly, proteins involved in cellular transport and trafficking, cytoskeletal dynamics, and mitochondrial functions are represented by Vacuolar protein sorting 35 (VPS35), Tubulin polymerization promoting protein (TPPP), and ATPase cation transporting 13A2 (ATP13A2), respectively (Kaur et al., 2021).

Cluster 2 in the functional network analysis included proteins such as ABL proto-oncogene 1, non-receptor tyrosine kinase (ABL1), and epidermal growth factor receptor (EGFR), which are involved in signal transduction and kinase activity (Colicelli, 2010). Neurotrophic factors identified in this cluster include brain-derived neurotrophic factor (BDNF) (Zuccato and Cattaneo, 2009). The calmodulin family, comprising calmodulin 3 (CALM3), calmodulin-like 3 (CALML3), calmodulin-like 4 (CALML4), calmodulin-like 5 (CALML5), and calmodulin-like 6 (CALML6), is associated with calcium signaling (Wang et al., 2019). Proteins involved in metabolism and energy production include glyceraldehyde-3-phosphate dehydrogenase (GAPDH), insulin (INS), and islet amyloid polypeptide (IAPP) (Abedini et al., 2018). Toll-like receptor 2 (TLR2) and toll-like receptor 4 (TLR4) play major roles in the immune response (Zacharowski et al., 2006). Finally, proteins involved in critical functions such as epigenetic regulation, protein degradation, the ubiquitin-proteasome system, nucleic acid binding and processing, synaptic function, and neurotransmission include histone deacetylase 6 (HDAC6), STIP1 homology and U-box containing protein 1 (STUB1), nucleolin (NCL), and neurogranin (NRGN) (Hwang et al., 2021; Seidel et al., 2015; Zhang et al., 2021).

Cluster 3 consisted of two proteins, SNAP25 (Synaptosome Associated Protein 25) and SYN1 (Synapsin I), which are involved in synaptic function and neurotransmitter release (Margiotta, 2021). Clusters 4, 5, 6, 7, and 8 are each associated with a single protein: SLC6A2 (Solute Carrier Family 6 Member 2), SLC1A2 (Solute Carrier Family 1 Member 2), NR4A2 (Nuclear Receptor Subfamily 4 Group A Member 2), SNCB (Beta-Synuclein), and RAB1A (Ras-related protein Rab-1A), respectively. These proteins are crucial for various roles, such as neurotransmitter transport, regulation of gene expression, synaptic function, neurodegenerative disorders, and intracellular vesicle trafficking and secretion (McCoy et al., 2015; Goldenring, 2013; Hu et al., 2020).

**FIGURE 4:**
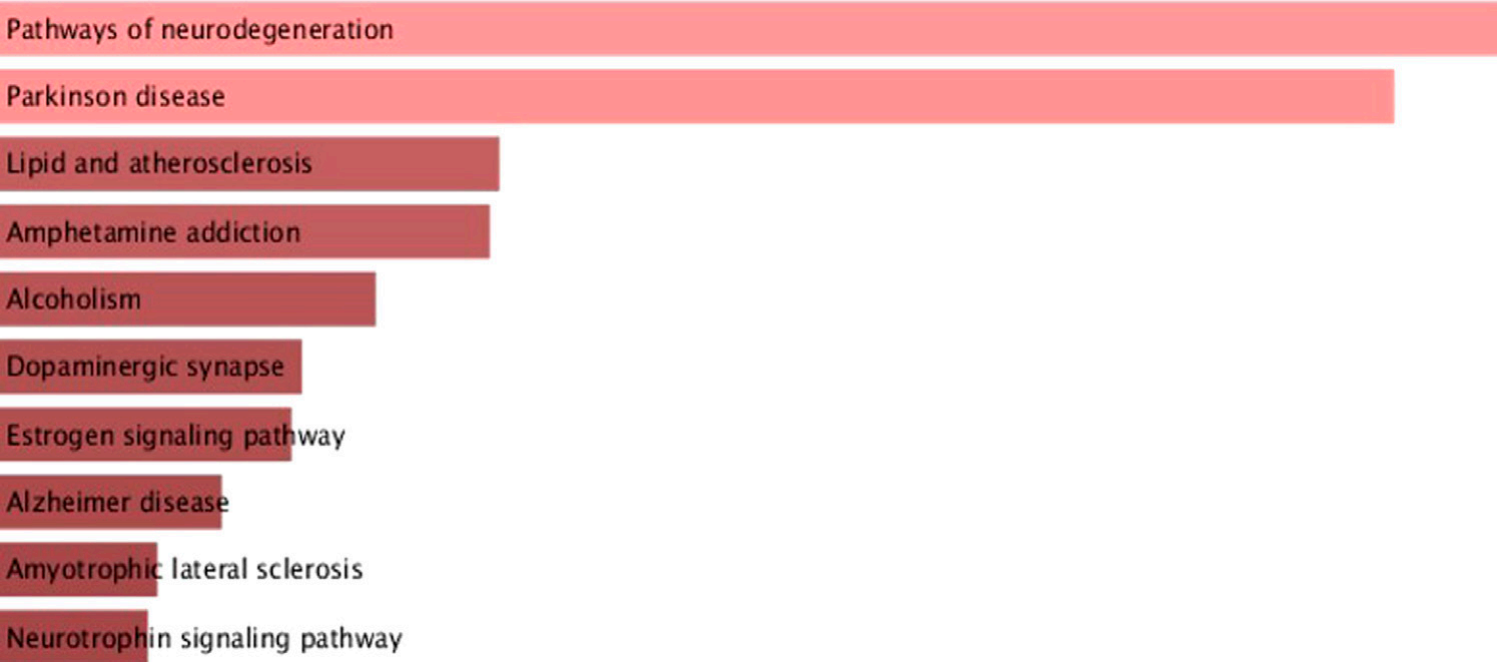
Pathway enrichment analysis of α-syn interactors substantiates α-syn’s involvement in neurodegenerative signaling and metabolic pathways.

In addition to the functional network analysis with different clusters, pathway enrichment analysis of *α*-syn and its interactors was conducted using the Enrichr database. The top 10 pathways involving *α*-syn and its interactors are highlighted in Figure 4, with *p*-values and adjusted *p*-values provided in Supplementary Table S2. *α*-syn interactors predominantly play significant roles in pathways related to neurodegeneration, PD, lipid and atherosclerosis, amphetamine addiction, alcoholism, dopaminergic synapses, estrogen signaling pathway, AD, amyotrophic lateral sclerosis, and the neurotrophin signaling pathway.

### 3.2 Molecular docking and interaction analysis

**FIGURE 5:**
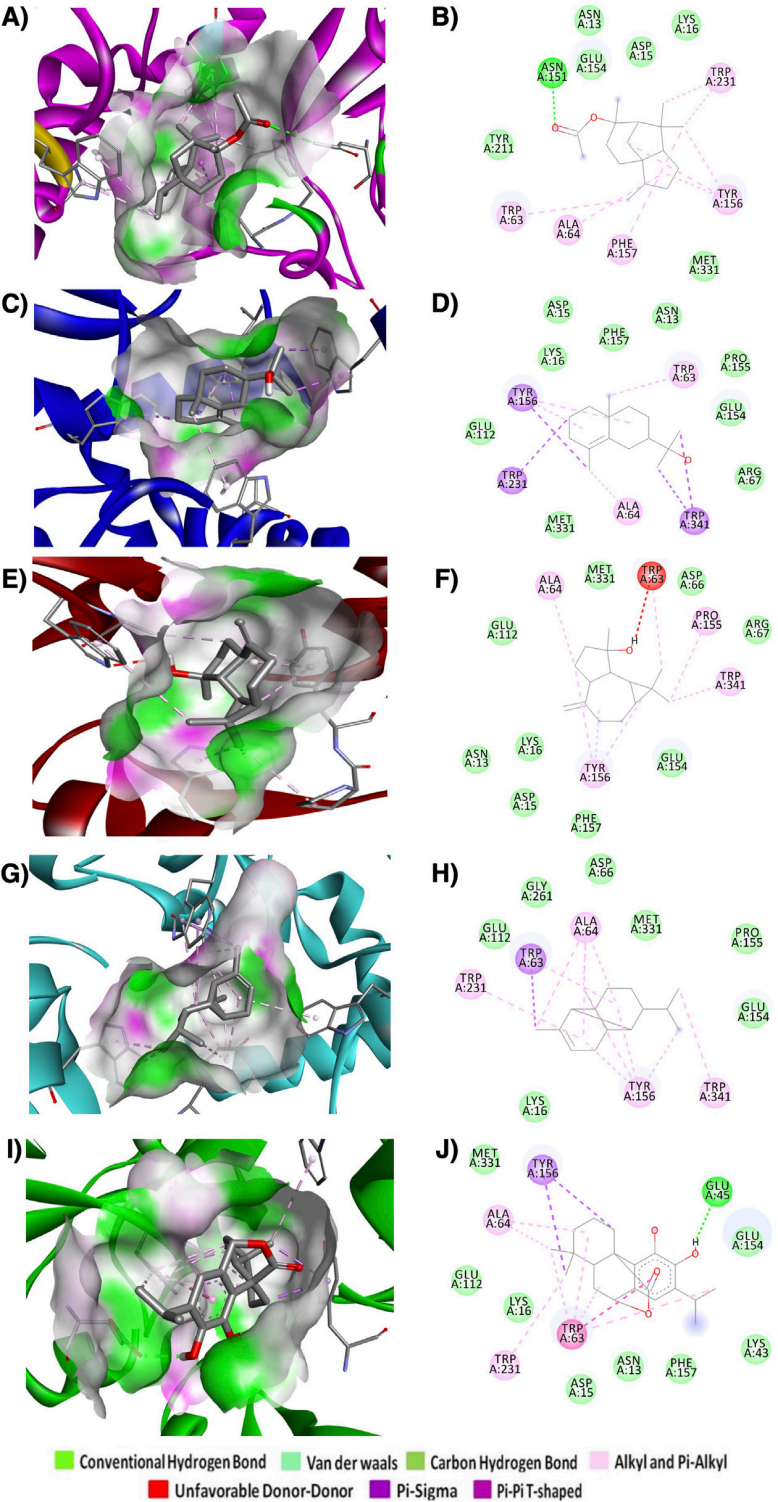
3D and 2D interactions of best docked ligands with *α*-syn. **(A)** and **(B)** cedryl acetate, **(C)** and **(D)** *γ*-eudesmol, **(E)** and **(F)** spathulenol, **(G)** and **(H)** *α*-copaene, **(I)** and **(J)** carnosol.

To identify the potential *α*-syn inhibitor, molecular docking was performed with 85 phytochemical compounds (Supplementary Table S3). These selected compounds have previously reported therapeutic potential such as antioxidant, antimicrobial, and anti-inflammatory activity (Nieto, 2017; Ramos da Silva et al., 2021). The binding energies of all selected compounds ranged from −4.2 kcal/mol to −11.4 kcal/mol. Among 85 compounds docked, the top 5 compounds were selected based on the top hit criteria of binding energies and BBB permeability and then further analysed for molecular interactions with *α*-syn (Figure 5; Table 1). The top hit compounds are *α*-copaene, carnosol, cedryl acetate, *γ*-eudesmol, spathulenol. Carnosol demonstrated a strong binding affinity for *α*-syn, with a binding energy of −9.4 kcal/mol. It formed a hydrogen bond of 4.82 Å with the A chain of Glu45 and exhibited other interactions with the A chains of Ala64, Trp63, Trp231, and Tyr156 (Figures 5I,J). Spathulenol showed −9.1 kcal/mol binding energy against *α*-syn (Figures 5E,F). No hydrogen bond was found to be involved in the interaction, but other interactions, such as unfavorable Donor-Donor interactions and two 2 groups of hydrophobic interactions (Alkyl and Pi Alkyl) with A chains of Ala64, Pro155, Trp63, Trp341, and Tyr156 were found to be involved. *γ*-eudesmol showed a high binding affinity towards *α*-syn with a binding energy of −8.9 kcal/mol. The interaction prediction study showed 1 group of electrostatic and 2 groups of hydrophobic interactions (Pi-Sigma, Alkyl, and Pi-Alkyl) with the A chains amino acids Ala64, Trp63, Trp231, Trp341, and Tyr156 (Figures 5C,D). Cedryl acetate and *α*-copaene showed a similar binding energy of −8.6 kcal/mol against *α*-syn. The molecular level interaction prediction study showed a hydrogen bond of length 5.16 Ǻ with the A chain residue Asn151 along with 2 groups of hydrophobic interactions (Alkyl and Pi-Alkyl) with Ala64, Phe157, Trp63, Trp231, and Tyr156 for cedryl acetate (Figures 5A,B). For *α*-copaene 1 group of electrostatic (Pi-Sigma) and 2 groups of hydrophobic interactions (Alkyl and Pi Alkyl) with the A chains amino acid residues Ala64, Trp63, Trp231, Trp341, and Tyr156 were found to be involved in the interactions (Figures 5G,H).

**TABLE 1:** H-bond and other interactions of selected compounds with α-syn.

Ligands	Binding free energy ΔG (kcal/mol)	No. of H-bonds	H-bonds and interacting residues	No. of other interactions	Other interactions and numbers	Other interaction and interacting residues
*α*-Copaene	−8.6	0	–	12	Pi-sigma (1), Alkyl and Pi-alkyl (11)	Ala64 (4), Trp63 (2), Trp231 (1), Trp341 (1), Tyr156 (4)
Carnosol	−9.4	1	Glu45	10	Pi-sigma (2), Pi-Pi T-shaped (1), Alkyl and Pi-alkyl (7)	Ala64 (2), Trp63 (5), Trp231 (1), Tyr156 (2)
Cedryl acetate	−8.6	1	Asn151	8	Alkyl and Pi-alkyl (8)	Ala64 (1), Phe157 (1), Trp63 (1), Trp231 (2), Tyr156 (3)
*γ*-Eudesmol	−8.9	0	–	8	Pi-sigma (4), Alkyl and Pi-alkyl (4)	Ala64 (1), Trp63 (1), Trp231 (1), Trp341 (2), Tyr156 (3)
Spathulenol	−9.1	0	–	8	Unfavorable Donor-Donor (1), Alkyl and Pi-alkyl (7)	Ala64 (1), Pro155 (1), Trp63 (2), Trp341 (1), Tyr156 (3)

### 3.3 BBB permeability analysis

**FIGURE 6:**
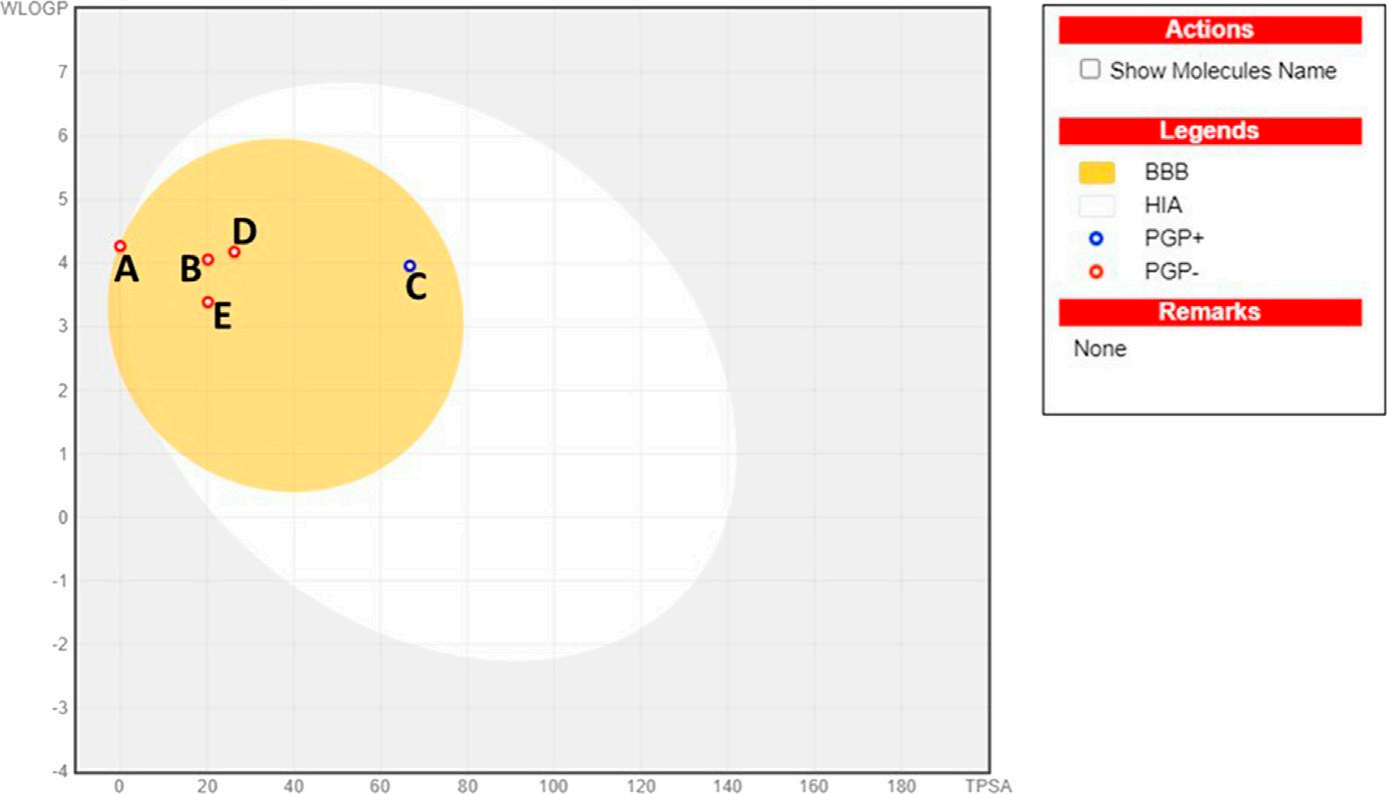
BBB permeability analysis using a boiled egg diagram for all top hit compounds. **(A)**- *α*-copaene, **(B)**- *γ*-eudesmol, **(C)**- carnosol, **(D)**-cedryl acetate, **(E)**- spathulenol.

Compounds’ BBB permeability was determined to understand their ability to penetrate the BBB to be available to the CNS. The BBB is a highly selective permeability barrier that maintains cerebral homeostasis by regulating the transport of nutrients and solutes into the CNS. This barrier presents a significant challenge for the delivery of therapeutics targeting CNS diseases, necessitating that CNS drugs possess the capability to traverse this dynamic and protective membrane (Upadhyay, 2014). The boiled egg diagram, plotting the TPSA against the partition coefficient (cLogP), can predict a molecule’s ability to cross the BBB and its potential for human intestinal absorption (HIA). The egg yolk represents the ability to traverse the BBB, while the egg white (albumin) signifies HIA. Our results suggested that all five compounds can penetrate the BBB to reach the CNS (Figure 6). Further, the BBB score from the molSoft web server confirmed the BBB permeability of all compounds, where a score of 6 indicates the highest permeability and 0 is the lowest (Table 2). Our results indicated that cedryl acetate had the highest BBB permeability, with a score of 4.53, followed by *γ*-eudesmol (4.39), spathulenol (4.39), and carnosol (3.84). In contrast, *α*-copaene showed borderline permeability with a score of 1.26, corresponding to our boiled egg diagram results.

**TABLE 2:** Drug likeness properties and BBB score.

Compounds	Lipinski Ro5	Molecular weight (g/mol)	HBA	HBD	cLogP	TPSA (Å^2^)	BBB score	Bioavailability score
*α*-Copaene	Yes; 1 violation: MLOGP>4.15	204.35	0	0	4.30	0.00	1.26	0.55
Carnosol	Yes; 0 violation	330.42	4	2	4.2869	66.76	3.84	0.55
Cedryl acetate	Yes; 0 violation	264.40	2	0	3.98	26.30	4.53	0.55
*γ*-Eudesmol	Yes; 0 violation	222.37	1	1	3.60	20.23	4.39	0.55
Spathulenol	Yes; 0 violation	220.35	1	1	3.26	20.23	4.39	0.55

### 3.4 Drug likeness and bioactivity analysis

The drug-likeness of compounds is based on Lipinski’s rule of five (Ro5: HBDs ≤5, HBA≤ 10, MW < 500, LogP ≤5, and no more than one violation), which comprises key molecular properties that affect a drug’s pharmacokinetics in the human body, including absorption, distribution, metabolism, and excretion (ADME) components (Lipinski et al., 1997).

Our results indicated that out of five compounds, *α*-copaene violates one of Lipinski’s rules as they have a slightly higher logP value greater than 4.15. As a rule, it does not predict pharmacological activity. However, the previously established anti-inflammatory and antioxidant activities should not be overlooked (Acharya et al., 2021; Nascimento et al., 2018; Poeckel et al., 2008; Ramos da Silva et al., 2021). Except for *α*-copaene, the other four compounds fulfill Lipinski’s Ro5, indicating their potential as active drug candidates (Table 2). Calculating the topological polar surface area (TPSA) provides insights into a drug’s bioavailability and hydrogen bonding potential. All of our compounds have a TPSA range from 0.00 to 66.76 Å (Table 2), lower than the typical upper limit of 160 Å (Abdelrheem et al., 2021). Furthermore, our results indicated an ideal bioavailability score of 0.55 for all tested compounds, suggesting good absorption in the human body (Martin, 2005).

Bioactivity scores for drug compounds can be evaluated based on parameters including kinase inhibition (KI), protease inhibition (PI), enzyme inhibition (EI), binding affinity to G protein-coupled receptors (GPCRs) and nuclear receptors (NRL), as well as ion channel modulation (ICM). A molecule with bioactivity scores above 0.00 is likely to exhibit significant biological activity; scores ranging from −0.50 to 0.00 are considered moderately active, while those below −0.50 are presumed to be inactive (Abdelrheem et al., 2021). All the bioactivity results were tabulated in Table 3. Our results indicated that α-copaene is highly active towards ICM, NRL, and EI and moderately active towards GPCRs and PI. Carnosol is predicted as highly active towards GPCRs, ICM, NRL, and EI and showed moderate activity for KI and PI. Cedryl acetate showed higher activity for GPCRs, ICM, NRL, and EI and moderate activity for PI but remained inactive towards KI. Similarly, γ-Eudesmol showed inactivity towards KI but was highly active towards ICM, NRL, and EI and moderately active for GPCRs and PI. Spathulenol exhibited high activity for NRL and EI and moderately active for GPCRs, ICM, and PI but inactive towards KI.

**TABLE 3:** Prediction of bioactivity score of compounds.

Compounds	GPCRs	ICM	KI	NRL	PI	EI
*α*-Copaene	−0.33	0.17	−0.79	0.02	−0.49	0.1
Carnosol	0.52	0.13	−0.26	0.51	−0.08	0.37
Cedryl acetate	0.01	0.25	−0.65	0.15	−0.13	0.61
*γ*-Eudesmol	−0.29	0.2	−0.81	0.53	−0.32	0.4
Spathulenol	−0.42	−0.28	−0.68	0.28	−0.36	0.06

### 3.5 Toxicity prediction

The toxicity of the drug compounds was assessed using AMES test results, human maximum tolerated dose, oral rat acute toxicity, hepatotoxicity, skin sensitization, minnow toxicity, and human ether-a-go-go gene (hERG) inhibition (Table 4). The AMES results indicated that all tested compounds are non-mutagenic and non-carcinogenic. The Maximum Recommended Tolerance Dose (MRTD) estimates human toxic doses, which are considered low if below log 0.477 (mg/kg/day) (Saha et al., 2021). Hence, the results obtained indicate that all tested compounds are less toxic to humans. The results also showed that none of our tested compounds are non-hERG inhibitors. Cedryl acetate, *γ*-eudesmol, and spathulenol showed skin sensitivity; however, no hepatotoxic prediction was established for all compounds. According to a report, a higher oral rat-acute toxicity (LD_50_) indicates less lethality compared to one with a lower LD_50_ value (Sinha et al., 2022). Our results indicate higher LD_50_ values ranging from 1.644 to 2.192, indicating less lethality for these compounds. However, if a molecule’s log LC_50_ (concentration causing 50% fathead minnow mortality) is below 0.5 mM (log LC_50_ < −0.3), it is considered highly toxic (Sinha et al., 2022). Our findings suggest that all the tested compounds except carnosol are less toxic, with significantly higher scores than the mentioned LC_50_ threshold. These results suggest that these five phytochemical compounds are less toxic to humans and can be potential drug candidates for treating CNS disorders.

**TABLE 4:** Toxicity prediction of compounds through ADMET pharmacokinetic properties.

Toxicity parameter	*α*-copaene	Carnosol	Cedryl acetate	*γ*-eudesmol	Spathulenol
AMES toxicity	No	No	No	No	No
Max. tolerated dose (human) (log mg/kg/day)	−0.302	0.227	−0.207	0.055	0.077
hERG I inhibitor	No	No	No	No	No
hERG II inhibitor	No	No	No	No	No
Oral Rat Acute Toxicity (LD50) (mol/kg)	1.644	2.192	1.646	1.681	1.687
Hepatotoxicity	No	No	No	No	No
Skin Sensitisation	No	No	Yes	Yes	Yes
Minnow toxicity (log mM)	0.128	−0.636	0.525	0.842	1.266

### 3.6 Molecular dynamic (MD) simulation analysis

**FIGURE 7:**
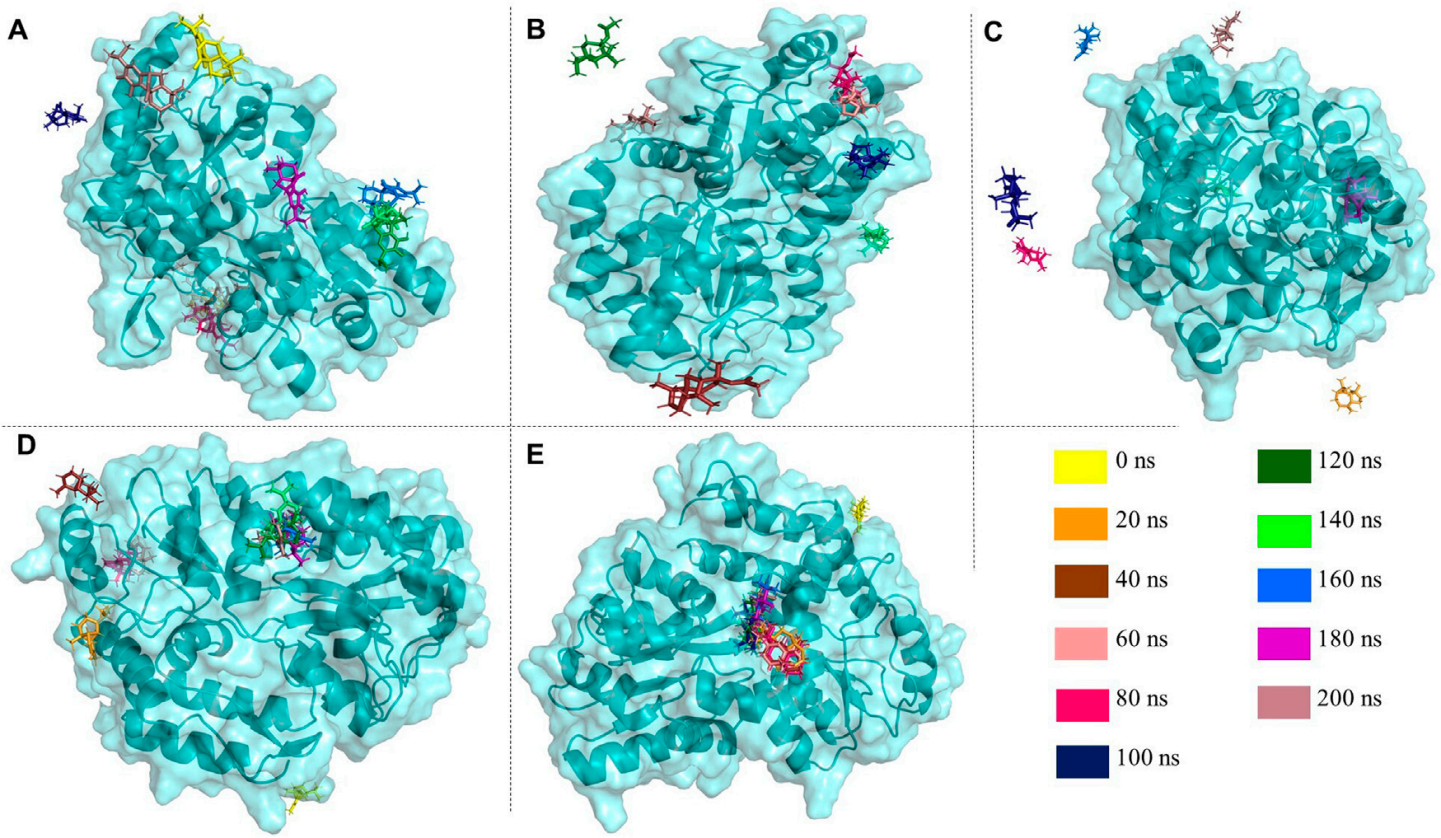
Various image panels extracted from PyMOL™ 2.5.8 illustrate the superimposition of the selected drugs. **(A)** carnosol **(B)** cedryl acetate **(C)** spathulenol **(D)** α-copaene **(E)** *γ*-eudesmol upon the targeted protein (PDB entry 3Q26) in the time-dependent manner of 20 ns interval throughout 200 ns to observe the concomitant protein-drug interactions.

Following a consecutive 200 ns molecular dynamics (MD) simulation, post-MD analysis was conducted to investigate the characteristic trajectories of the reference protein-only system and the respective protein-ligand complexes. This analysis involved the examination of various trajectory characteristics, including root mean square deviation (RMSD), root mean square fluctuation (RMSF), radius of gyration (R_g_), solvent accessible surface area (SASA), hydrogen bond (HB) occupancy, and pair distance. These metrics were utilized to evaluate protein-ligand interactions and assess the protein’s stability and folding behavior. The superimposition of ligands on the targeted proteins was shown in Figure 7 to observe the time-dependent protein-drug interaction.

#### 3.6.1 RMSD and backbone stability analysis

**FIGURE 8:**
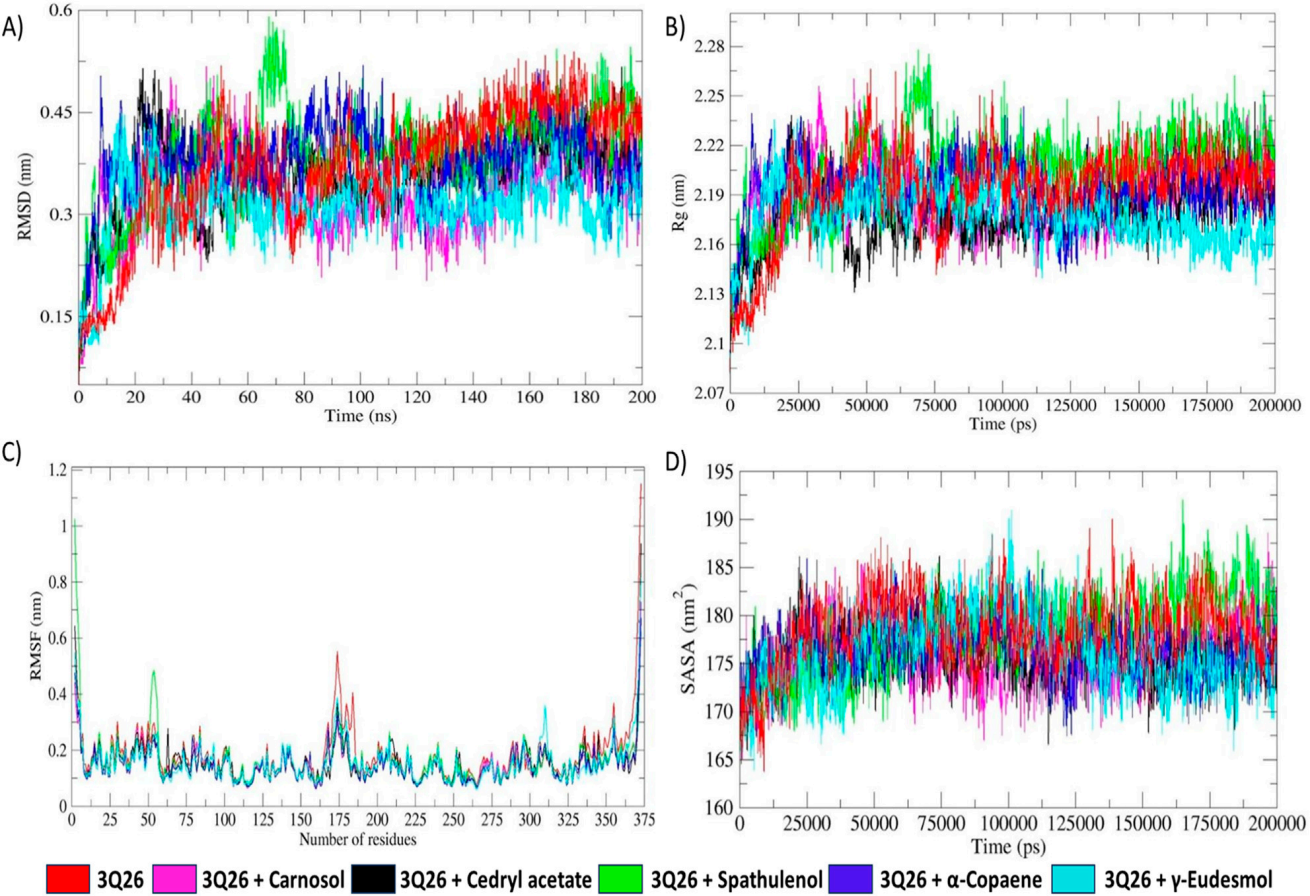
**(A)** RMSD (root mean square deviation) plot of protein backbone fluctuations, **(B)** comparative analysis plot of R_g_ (radius of gyration) fluctuations, **(C)** RMSF (root mean square fluctuation) plot of residual fluctuations, **(D)** comparative analysis plot of SASA (solvent accessible surface area) profiles of protein-only system and protein-docked complexes depicted in specific colour codes.

Figure 8A presents the superimposition of all RMSD plots, providing an approximate overview of the significant overlap in the RMSD trajectories of the reference protein-only system and the protein-ligand complexes. This indicates minimal deflection in backbone stability following ligand association. The RMSD, which reflects backbone stability, corroborates the altered protein folding behavior, as validated by the respective R_g_ trajectories (García and Onuchic, 2003). The concordant fluctuations observed in the R_g_ and RMSD plots further describe the folding behaviors of the respective systems.

Individual RMSD plots for all systems show minimal differences in fluctuations, ranging from approximately 0.1–0.55 nm, as depicted in Figure 8. As illustrated in Figure 8, the reference protein-only system (3Q26) and other protein-ligand complexes exhibited erratic RMS deviations up to approximately 20 ns, indicating initial backbone instability. This was followed by stabilization, suggesting a rapid folding process toward the native and thermodynamically favorable conformation (García and Onuchic, 2003; Maruyama et al., 2023). Protein folding is primarily influenced by the interplay of solvation and nonpolar-solvation (de-solvation) effects (García and Onuchic, 2003). Thus, it can be hypothesized that the initial 20 ns interval of erratic RMSD fluctuation observed across all RMSD trajectories is predominantly driven by the solvation effect mediated by TIP3P water molecules in the MD environment. This solvation effect is common to all RMSD profiles. In contrast, the RMSD fluctuations observed in the latter intervals for protein-ligand systems are critically influenced by the de-solvation effect due to ligand interactions.

#### 3.6.2 Radius of gyration (R_g_) and molecular compactness analysis

The radius of gyration (R_g_), which signifies molecular and structural compactness, can be calculated as:

$$R_{g}=\sqrt{\frac{1}{N}\sum_{i=1}^{N}{\left(r_{i}-r_{com}\right)}^{2}}$$

Where *N* is the total number of atoms, *r*_*com*_ is the position vector at the centre of mass and *r*_*i*_ is the position vector at the *i*th atom of respective protein macromolecules (Bronowska, 2011). R_g_ is a fundamental metric for quantifying the spatial distribution of atoms around the centre of mass, thereby evaluating structural integrity and compactness. As depicted in Figure 8B, the R_g_ trajectories showed higher fluctuations up to approximately 20 ns for both the protein-only and protein-docked systems, likely due to the solvation effect, similar to the RMSD fluctuations.

As time progressed, the 3Q26-*γ*-eudesmol complex maintained lower R_g_ fluctuations around 2.16 nm, while the 3Q26 and 3Q26-spathulenol systems showed higher R_g_ fluctuations around 2.23 nm. This suggests that the 3Q26-*γ*-eudesmol complex exhibited more stable protein folding, achieving the native, thermodynamically favored conformation more effectively than the 3Q26 and 3Q26-spathulenol systems.

For specific protein-ligand complexes, such as 3Q26-spathulenol and 3Q26-α-copaene, intermittent unsteady R_g_ fluctuations were observed during the intervals of approximately 60–83 ns and 90–126 ns. These fluctuations illustrate that the protein tends to optimize its feasible native conformation due to successive ligand accommodations, resulting in slight adjustments to structural integrity as reflected in the R_g_ trajectories.

#### 3.6.3 RMSF analysis

The root mean square fluctuation (RMSF) describes the fluctuation of amino acid residues in both protein-only and protein-ligand systems, as depicted in Figure 8C. RMSF is calculated based on the C_α_ atom fluctuations around their average positions, both in the presence and absence of ligand moieties. Residues with higher fluctuations indicate greater flexibility, while those with lower fluctuations suggest increased rigidity, influenced by weakening or strengthening ligand interactions, respectively (Chaudhary and Aparoy, 2017).

As shown in Figure 10, the protein-only system exhibits significantly higher residue flexibilities in the regions spanning the 165–185th and 334–375th residues, indicative of increased structural instability compared to the protein-ligand systems. This instability is further corroborated by the reduced compactness observed in the protein-only system, confirmed by the less stable radius of the gyration (R_g_) trajectory (Ghahremanian et al., 2022). In contrast, the protein-ligand systems demonstrate enhanced stability and structural integrity in their R_g_ trajectories, supporting the consistency in RMSF residual rigidity throughout the MD simulation.

**FIGURE 9:**
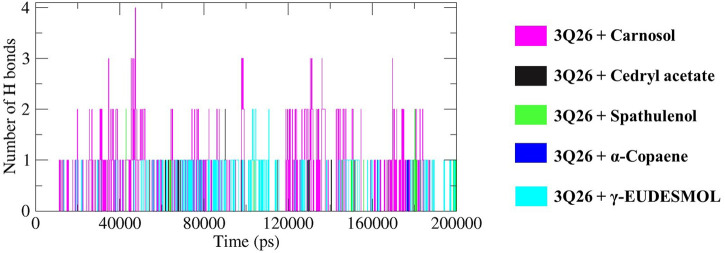
Illustration of hydrogen bond (HB) occupancy contributed by the interaction between protein and respective docked ligands depicted in specific color codes.

Among all ligand-docked systems, the 3Q26-spathulenol complex exhibits comparatively higher residue flexibility, particularly in the residues spanning 50–57. This suggests reduced hydrogen bond (HB) formation and the lowest electrostatic energy decomposition, as shown in Figure 9. Other protein-ligand complexes, including 3Q26-carnosol, 3Q26-cedryl acetate, 3Q26-*α*-copaene, and 3Q26-*γ*-eudesmol, display significantly constrained residue fluctuations, ranging from approximately 0.06–0.38 nm. This underscores their augmented electrostatic interactions and evident HB occupancy, as indicated in Figure 9.

#### 3.6.4 SASA examination

Solvent accessible surface area (SASA) is a critical metric for assessing hydrophobic interactions, often overestimating the effects when modeled explicitly, particularly with encapsulated solvation domains and hydrophobic non-polar molecules (Eisenhaber, 1999). The solvation model rendered by TIP3P water molecules and the nonpolar solvation effect exerted by docked ligand moieties significantly impact protein folding and resultant energy dissipation (Ausaf Ali et al., 2014). A gradual decrease in SASA trajectory indicates slow, stable protein folding dynamics influenced by stronger hydrophobic forces, whereas an increase in SASA values suggests rapid, unstable folding driven by weaker hydrophobic interactions (Duan et al., 2013).

The SASA trajectory plot in Figure 8D reveals that the protein-only system (3Q26) and the protein-docked system with spathulenol exhibit slightly higher SASA values (∼183 nm^2^). In contrast, other protein-ligand complexes (3Q26-carnosol, 3Q26-cedryl acetate, 3Q26-*γ*-eudesmol, and 3Q26-α-copaene) show a significant decrease in average SASA values (∼173 nm^2^). These SASA trajectories highlight distinct protein folding behaviors for the 3Q26 protein with various ligands. Specifically, the 3Q26 and 3Q26-spathulenol systems exhibit rapid and unstable folding patterns, indicative of reduced hydrophobic interactions. Conversely, the 3Q26 complexes with carnosol, cedryl acetate, *γ*-eudesmol, and α-copaene demonstrate more stable and slower folding dynamics, influenced by stronger hydrophobic interactions, as reflected by reduced SASA fluctuations.

#### 3.6.5 Hydrogen bond (HB) formation and subsequent pair distance analysis

As illustrated in Figure 9, the hydrogen bond (HB) occupancy plot for the 3Q26-docked complexes is dominated by carnosol, followed by *γ*-eudesmol, cedryl acetate, α-copaene, and spathulenol. This trend aligns with the decreasing order of electrostatic energy dissipation (kJ/mol) shown in Table 5.

**TABLE 5:** MM-PBSA (Molecular Mechanics Poisson-Boltzmann Surface Area) based approximate binding free energy (KJ/mol) calculation of top 5 selected small molecules against the reference macromolecule alpha-synuclein chimeric protein (PDB entry 3Q26).

Drugs	Van der waals energy (KJ/mol)	Electrostatic energy (KJ/mol)	Polar solvation energy (KJ/mol)	SASA energy (KJ/mol)	Binding free energy (KJ/mol)
Carnosol	−57.564	−11.084	42.464	−7.722	−33.906
Cedryl acetate	−20.145	−2.345	23.960	−2.968	−1.498
Spathulenol	−7.799	−0.376	−22.024	−1.373	−31.572
*α*-Copaene	−58.857	−0.730	26.847	−8.127	−40.867
*γ*-Eudesmol	−90.186	−3.361	33.535	−11.055	−71.067

Carnosol exhibited regular intervals of HB formation, starting at the 10th ns with the formation of 1 HB, followed by 2, 3, and 4 HBs at the 20th, 34th, and 47th ns, respectively. The HB formation then decreased, with 3 and 2 HBs observed up to the 169th and 183rd ns, respectively. Similarly, *γ*-eudesmol showed consistent HB occupancy, starting with 1 and 2 HBs at the 10th and 103rd ns, respectively, and maintaining 1 HB up to the 200th ns.

**FIGURE 10:**
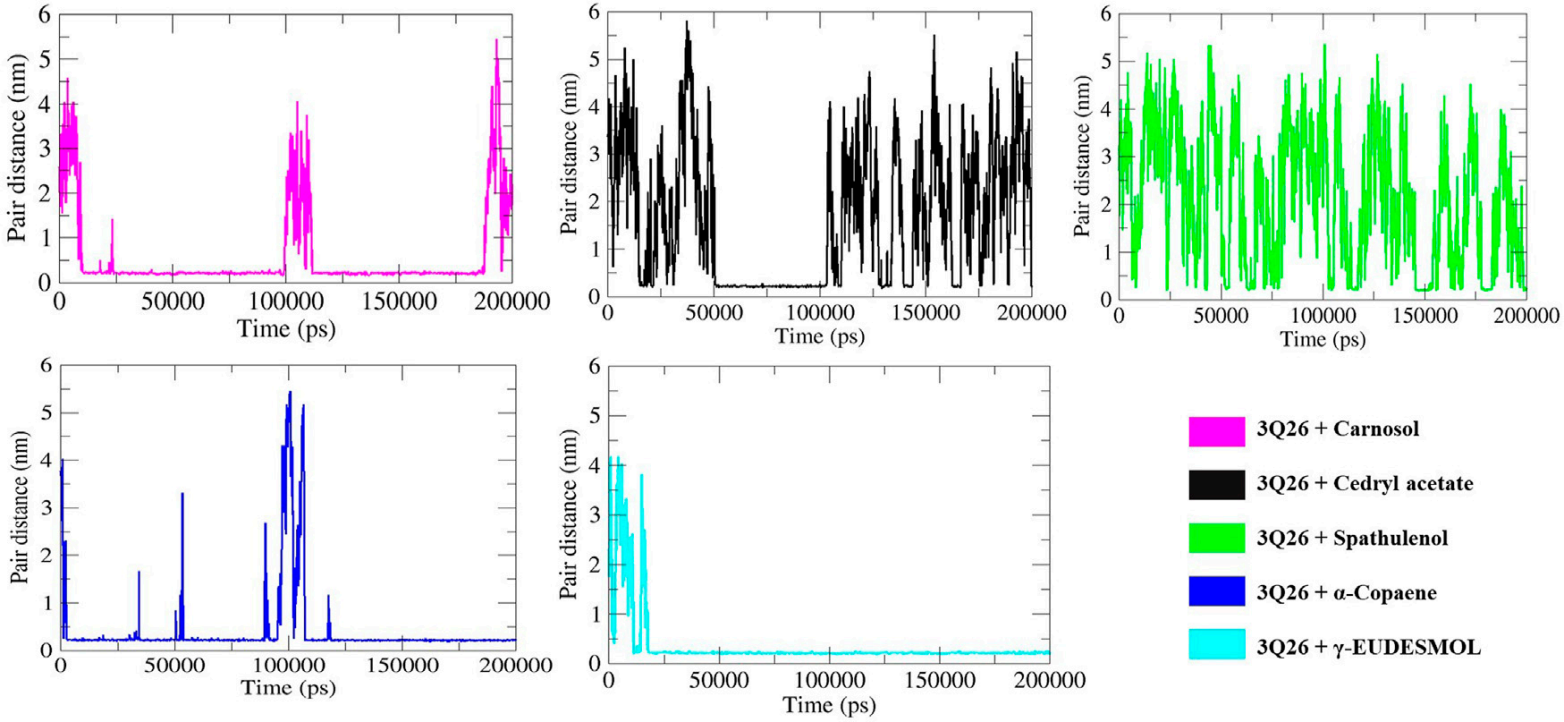
Pair distance (PD) analysis between five differential pairs of protein 3Q26 with specific docked ligands – Carnosol, cedryl acetate, spathulenol, α-copaene, and γ-eudesmol represented in respective panels by 5 separate color codes.

In contrast, cedryl acetate, *α*-copaene, and spathulenol displayed intermittent HB formation at specific intervals, as depicted in Figure 9. The pair distance analysis highlights the most stabilized interaction in the 3Q26-*γ*-eudesmol complex, as shown in panel F of Figure 10, where the proximity of protein 3Q26 and ligand *γ*-eudesmol is maintained throughout the MD interval of 20–200 ns. Following *γ*-eudesmol, carnosol maintained proximity during the MD intervals of 25–100 ns and 110–185 ns. Cedryl acetate maintained close pair distance at the MD interval of 50–100 ns. α-Copaene remained within the binding pockets of 3Q26 during the consecutive MD intervals of 5–50 ns, 55–90 ns, and 120–200 ns, constructing a successive interaction dynamic.

In contrast, spathulenol showed intermittent dynamics in maintaining proximity and stable fitting within the binding pocket of 3Q26, supporting its least occupancy in hydrogen bond formation and consequent minimal dissipation of electrostatic energy, as indicated in Table 5.

### 3.7 MM-PBSA binding free energy (kJ/mol) analysis

Binding free energy decomposition was evaluated based on the MM-PBSA method. The total binding free energy (ΔG_BA_) is the resultant combination of four segregated forms of energy: Van der Waals energy (kJ/mol), electrostatic energy (kJ/mol), polar solvation energy (kJ/mol), and SASA energy (kJ/mol).

Van der Waals energy represents weak interaction energy, primarily arising from permanent and transient hydrophobic, non-polar interaction forces (*π−σ*, *π*−alkyl, and *π−π*). In the MD environment, the de-solvation effect primarily contributes to the rise of van der Waals and electrostatic energy dissipation (Mahmoud et al., 2020; Rasaiah et al., 2008). Therefore, van der Waals forces are directly anti-correlated with polar solvation effects, which are effectively mediated by TIP3P water molecules (Naïm et al., 2007). In Table 5, van der Waals energy is highlighted as a major contributor to the ΔG_BA_ decomposition, except in the case of spathulenol interaction where the ΔG_BA_ is notably influenced by unfavorable polar solvation energy (∼−22.024 kJ/mol). This observation underscores a direct anti-correlation between polar solvation energy and van der Waals energy. Among the five different small molecule drugs studied, *γ*-eudesmol exhibits the highest dissipation in van der Waals energy (∼−90.186 kJ/mol), leading to the highest ΔG_BA_ decomposition (∼−71.067 kJ/mol) observed among all interactions.

Similarly, the compounds carnosol, cedryl acetate, and α-copaene show substantial van der Waals energy dissipation (approx. −57.564 kJ/mol, approx. −20.145 kJ/mol, approx. −58.857 kJ/mol, respectively), contributing significantly to their respective ΔG_BA_ values. These interactions illustrate how non-polar van der Waals interactions mitigate the polar solvation effect and promote de-solvation effects, thereby enhancing electrostatic interactions to a notable extent. De-solvation or non-polar solvation plays a crucial role in modulating electrostatic energy flow and subsequent HB formation (Dill et al., 2002). Therefore, the intensity of hydrogen bond (HB) formation (Figure 9) corresponds to the order of electrostatic energy dissipation observed in the interactions between 3Q26 and carnosol, cedryl acetate, spathulenol, *α*-copaene, and *γ*-eudesmol. The interaction of 3Q26 with carnosol, which dissipates the highest electrostatic energy (approx. −11.084 kJ/mol), exhibits the highest occupancy in HB formation. This is followed by *γ*-eudesmol (electrostatic energy approx. −3.361 kJ/mol), cedryl acetate (electrostatic energy approx. −2.345 kJ/mol), α-copaene (electrostatic energy approx. −0.730 kJ/mol), and spathulenol (electrostatic energy approx. −0.376 kJ/mol).

Polar solvation energy, regulated by the aqueous nature of the MD environment, significantly influences the interactions of drug molecules with macromolecules. Positive (+ve) polar solvation energy dissipation is favorable for enhancing electrostatic, hydrophobic, and van der Waals interactions, thereby improving the binding affinity of protein-ligand interactions. Conversely, negative (-ve) polar solvation energy dissipation hinders significant protein-ligand interactions and stable protein folding dynamics.

Spathulenol, which exhibits the highest negative polar solvation energy dissipation (approx. −22.024 kJ/mol) among the selected drugs, also shows the lowest dissipation of electrostatic and SASA (solvent accessible surface area) energy (approx.−1.373 kJ/mol). This correlates with its least occupancy in HB formation and the most unstable pair distance (Figure 10) throughout the 200 ns MD simulation interval.

## 4 Discussion

The present study investigated the role of the neuronal protein *α*-syn in neurodegenerative diseases using functional networking and pathway enrichment analysis. Additionally, it evaluated the neuroprotective potential of Lamiaceae family bioactive compounds against *α*-syn aggregation through molecular docking and simulation approach. The functional network and enrichment analysis demonstrated the association of *α*-syn with various proteins involved in NDs, which α-syn may directly or indirectly regulate. Additionally, *α*-syn was found to be involved in several neurodegenerative signaling pathways such as neurodegeneration, PD, lipid and atherosclerosis, amphetamine addiction, alcoholism, dopaminergic synapses, estrogen signaling pathway, AD, amyotrophic lateral sclerosis, and the neurotrophin signaling pathway, underscoring its impact on the initiation and progression of various NDs. It is well known that *α*-syn is associated with PD and is a potential molecular drug target for its therapeutic solutions (Horne et al., 2024). However, our findings suggest that *α*-syn is not only a potential target for PD but also a viable target for other ND therapeutics.

As *α*-syn is a potential target molecule for NDs, in this study we found five compounds such as *α*-copaene, carnosol, cedryl acetate, *γ*-eudesmol, and spathulenol that exhibited strong binding interactions with *α*-syn. Strong interactions of these five selected phytochemical compounds with *α*-syn indicate their potency as probable inhibitors. However, only a limited number of *α*-syn inhibitors have been identified to date, such as Anle138b, NPT200-11, SynuClean-D, ZPD-2, and CLR01 (De Luca et al., 2022; Fields et al., 2019). Consequently, there is a critical need for further development of *α*-syn inhibitors. Our findings suggest that these phytochemicals could serve as effective agents to prevent *α*-syn aggregation, enhance *α*-syn degradation, inhibit *α*-syn expression, or disrupt *α*-syn interactions with other proteins implicated in NDs. Moreover, *α*-copaene, carnosol, cedryl acetate, *γ*-eudesmol, and spathulenol have been identified as potent antioxidants and anti-inflammatory agents (Acharya et al., 2021; Nascimento et al., 2018; Poeckel et al., 2008; Ramos da Silva et al., 2021) that attenuate cellular oxidative stress and neural inflammation associated with various NDs, thereby acting as potential neuroprotective agents. These results underscore the potential of these compounds as promising candidates for *α*-syn-targeted drug development.

The BBB’s protective nature makes it challenging for drug molecules to cross, posing a major hurdle for CNS drug candidates that should be addressed early in drug discovery. Drugs targeting peripheral organs should also be checked for BBB permeability to prevent potential CNS side effects (Alavijeh et al., 2005). Therefore, our results elucidated the BBB permeation ability of these five phytochemical compounds, which can be further utilised as potential CNS drugs to combat various NDs. Depending on the docking analysis, further pharmacokinetic study was performed to analyze these top-hit compounds as potent drugs. The present investigation demonstrated that these compounds have excellent CNS bioavailability, lower toxicity levels, and significant biological activities.

Moreover, MD simulation analysis elucidated the interaction stability of these five compounds with *α*-syn. A precise examination of backbone stability during the MD interval of approximately 30–200 ns reveals that notable RMS deviations in the protein-ligand systems (PDB entry 3Q26 complexed with cedryl acetate, spathulenol, and *α*-Copaene) align closely with the RMSD trajectory of the protein-only system (3Q26 without ligand). This indicates that the aqueous environment-driven solvation effect mitigates the de-solvation effect, as the respective ligand interactions play a minor role. This is evidenced by lower hydrogen bond occupancy (Figure 9) and electrostatic energy decomposition. Therefore, this hypothesis supports the notion that the increased backbone fluctuations (RMSD ∼0.4 nm) in the 3Q26 complexes with cedryl acetate, spathulenol, and *α*-Copaene lead to faster and less stable protein folding.

Conversely, the dynamics of 3Q26 interactions with carnosol and *γ*-eudesmol demonstrate comparatively stable backbone fluctuations, indicating a slower, more stable protein folding behavior. This stability is due to the significant influence of ligand interactions and the prevailing solvation effect, where ligand-mediated de-solvation and electrostatic interactions dominate over the solvation effect, as observed in the hydrogen bond occupancy plot. Similarly, A stable R_g_ trajectory indicates a well-packed protein conformation, with minimal fluctuations suggesting retention of the native fold. In contrast, an unfolded protein shows a higher, unsteady R_g_. R_g_ and RMSD plots often correlate, offering insights into how structural compactness and backbone flexibility affect protein-ligand interactions (Kim et al., 2015; Rosilan et al., 2024; Yamamoto et al., 2021). The R_g_ values of proteins and protein-ligand complexes reflect the compactness and structural integrity of proteins in the presence of the tested compounds. Simultaneously, comprehensive SASA analysis aligns with RMSD and R_g_ results, validating the observed protein folding dynamics. The SASA data also delineate the simultaneous interplay of solvation and de-solvation effects in stabilizing protein-ligand interactions, providing a holistic view of SASA and the protein-folding pathways. The HB formation between protein-ligand indicates short-range electrostatic interactions occurring through intra- or intermolecular covalent bonds between hydrogen and electronegative atom(s). Intermolecular HB formation strengthens the stabilized ligand-protein interaction, resulting in significant electrostatic energy dissipation, as detailed in Table 5 of the binding free energy decomposition (Choudhary et al., 2023). In protein-ligand interaction dynamics, the pair distance defines the specific MD intervals during which the ligand stays within the protein’s binding pocket. Pair distance also illustrates the role of interacting forces in driving protein-ligand interactions, as shown in the HB occupancy plot in Figure 9 (Kandasamy et al., 2022). At the same time, SASA energy, influenced by the hydrophobicity and amphiphilicity of the ligands, plays a crucial role in determining the effective binding affinity and protein folding dynamics of protein-ligand complexes (Eisenberg and McLachlan, 1986). Table 5 shows that the van der Waals, electrostatic, and SASA energy of different protein-ligand complexes exhibit a consistent increasing or decreasing order. This correlation underscores the intricate interplay between ligand-mediated and de-solvation effects in SASA energy decomposition and subsequent protein folding dynamics. Among all five interactions studied, the *γ*-eudesmol and spathulenol interactions with 3Q26 show maximum (approx. −11.055 kJ/mol) and minimum (approx.−1.373 kJ/mol) SASA energy flows, respectively. This validates their highest and lowest dominance in HB formation (Figure 9) and the most stable and unstable maintenance of pair distances (Figure 10, respectively). This pattern also supports the corresponding order of electrostatic energy dissipation in these interactions. Ultimately, it can be postulated that the backbone stability, hydrophobic/hydrophilic surface nature of proteins, and subsequent altered protein folding dynamics of native proteins are significantly influenced by ligand interactions. These interactions dictate the subsequent de-solvation and solvation effects, thereby affecting the substantial dynamics of ΔG_BA_ decomposition. Therefore, ΔG_BA_ decomposition alone may not always indicate strong protein-ligand interactions, as the dissipation of van der Waals energy, SASA energy, and electrostatic energy can vary, reflecting the complex mechanisms of protein-ligand interactions challenged by the inherent solvation model of the MD environment.

Moreover, these findings suggest that *α*-copaene, *γ*-eudesmol, carnosol, cedryl acetate, and spathulenol have the potential to counteract *α*-syn as an alternative neuroprotective solution. However, phytochemical-based drug discovery using molecular docking and ADMET analysis offers a cost-effective approach to identify novel therapeutic compounds. However, *in silico* predictions are limited by structural data and may not accurately capture pharmacokinetics or toxicity. Based on the data presented, the compounds investigated in this study could be considered for further *in vitro*, *in vivo* experimental validation, and clinical studies for the potential treatment of NDs.

## Data Availability

The relevant information on docking for all tested compounds as discussed in the main text is provided in a separate archive at https://zenodo.org/records/13192341. Software used in this manuscript are third-party software and packages; versions are described in detail in the Materials and Methods section. All the data are provided in the supplementary file and above mentioned link. Further inquiries can be directed to the corresponding author(s).

## Abbreviations

NDs, Neurodegenerative Disorders; CNS, Central Nervous System; PNS, Peripheral Nervous System; DALYs, Disability Adjusted Life Years; AD, Alzheimer’s Disease; PD, Parkinson’s Disease; HD, Huntington’s Disease; ADMET, Absorption, Distribution, Metabolism, Excretion, Toxicity; α-syn, α-synuclein; ROS, Reactive Oxygen Species.
