# Supplementary material for: Repurposing major metabolites of lamiaceae family as potential inhibitors of α-synuclein aggregation to alleviate neurodegenerative diseases: an in silico approach
Source: Front Pharmacol. 2025 Apr 16;16:1519145. doi: 10.3389/fphar.2025.1519145 (PMC12041775; doi:10.3389/fphar.2025.1519145)
Supplement: Supplementary file 1 [file Table1.docx]

**Repurposing Major Metabolites of Lamiaceae Family as Potential Inhibitors of *α*-Synuclein** **Aggregation to Alleviate Neurodegenerative Diseases: An *In Silico* Approach**

Soham Bhattacharya^1^, Neha Gupta^2^, Adrish Dutta^2^, Pijush Kanti Khanra^3^, Ritesh Dutta^4^, Jana Žiarovská^5^, Nikolay T. Tzvetkov^6^, Lucie Severová^7^, Lenka Kopecká^7^, Luigi Milella^8*^, Eloy Fernández-Cusimamani^2^

^1^Department of Agroecology and Crop Production, Faculty of Agrobiology, Food and Natural Resources, Czech University of Life Sciences Prague, Kamýcká 129, Prague 6 – Suchdol, 165 00, Czech Republic.

^2^Department of Crop Sciences and Agroforestry, Faculty of Tropical AgriSciences, Czech University of Life Sciences Prague, Kamýcká 129, Suchdol, 165 00 Prague 6, Czech Republic.

^3^Department of Biosciences and Bioengineering, Indian Institute of Technology Guwahati, Guwahati, 39, Assam, India

^4^Environmental Biotechnology & Genomics Division, CSIR-National Environmental Engineering Research Institute (CSIR-NEERI), Nehru Marg, Nagpur 440020, India

^5^Institute of Plant and Environmental Sciences, Faculty of Agrobiology and Food Resources, Slovak University of Agriculture in Nitra, Tr. A. Hlinku 2, Nitra, Slovak Republic

^6^Department of Biochemical Pharmacology & Drug Design, Institute of Molecular Biology “Roumen Tsanev”, Bulgarian Academy of Sciences (BAS), Acad. G. Bonchev Str., bl. 21, 1113 Sofia, Bulgaria

7Department of Economic Theories, Faculty of Economics and Management, Czech University of Life Sciences Prague, Kamýcká 129, 165 00 Prague, Czech Republic

^8^Department of Science, University of Basilicata, V.le Ateneo Lucano 10, 85100 Potenza, Italy

*Address correspondence to:

Luigi Milella

Email: [luigi.milella@unibas.it](mailto:luigi.milella@unibas.it), Phone/fax +390971205525

Table S1. Gene name, protein identifier of α-syn and its interactors

| **Gene name** | **protein identifier** | **Protein name** |
| --- | --- | --- |
| APOA1 | 9606.ENSP00000236850 | Apolipoprotein AI |
| APOE | 9606.ENSP00000252486 | Apolipoprotein E |
| APP | 9606.ENSP00000284981 | Amyloid beta precursor protein |
| ATP13A2 | 9606.ENSP00000327214 | ATPase cation transporting 13A2 |
| ATXN3 | 9606.ENSP00000496695 | ataxin 3 |
| BCL2L1 | 9606.ENSP00000365230 | proteins BCL2 like 1 |
| CLU | 9606.ENSP00000315130 | Clusterin |
| CTSD | 9606.ENSP00000236671 | Cathepsin D |
| FBXO7 | 9606.ENSP00000266087 | F-box protein 7 |
| FYN | 9606.ENSP00000346671 | Fyn proto-oncogene, Src family tyrosine kinase |
| GBA | 9606.ENSP00000314508 | Lysosomal acid glucosylceramidase |
| GIGYF2 | 9606.ENSP00000387170 | GRB10 interacting GYF protein 2 |
| GSN | 9606.ENSP00000362924 | Gelsolin |
| HSPA9 | 9606.ENSP00000297185 | Heat shock protein family A member 9 |
| HTRA2 | 9606.ENSP00000258080 | HtrA serine peptidase 2 |
| HTT | 9606.ENSP00000347184 | Huntingtin |
| KLK6 | 9606.ENSP00000366047 | Kallikrein-6 |
| LRRK2 | 9606.ENSP00000298910 | Leucine-rich repeat kinase 2 |
| MAOA | 9606.ENSP00000340684 | Monoamine oxidase A |
| MAOB | 9606.ENSP00000367309 | Monoamine oxidase B |
| MAPT | 9606.ENSP00000340820 | Microtubule-associated protein tau |
| NEDD4 | 9606.ENSP00000424827 | Neural precursor cell expressed developmentally down-regulated protein 4 |
| PARK7 | 9606.ENSP00000340278 | Parkinsonism-associated deglycase |
| PINK1 | 9606.ENSP00000364204 | PTEN-induced putative kinase 1 |
| PRKN | 9606.ENSP00000355865 | Parkin RBR E3 ubiquitin-protein ligase |
| PRNP | 9606.ENSP00000399376 | Prion protein |
| PSEN1 | 9606.ENSP00000326366 | Presenilin-1 |
| PSMC1 | 9606.ENSP00000261303 | Proteasome 26S subunit, ATPase1 |
| SLC18A1 | 9606.ENSP00000387549 | Vesicular monoamine transporter 1 |
| SLC18A2 | 9606.ENSP00000496339 | Vesicular monoamine transporter 2 |
| SLC6A3 | 9606.ENSP00000270349 | Dopamine transporter |
| SNCAIP | 9606.ENSP00000261367 | Synuclein alpha interacting protein |
| SOD1 | 9606.ENSP00000270142 | Superoxide dismutase 1 |
| SQSTM1 | 9606.ENSP00000374455 | Sequestosome 1 |
| TARDBP | 9606.ENSP00000240185 | TAR DNA binding protein |
| TPPP | 9606.ENSP00000353785 | Tubulin polymerization promoting protein |
| TTR | 9606.ENSP00000237014 | Transthyretin |
| UBB | 9606.ENSP00000478771 | Ubiquitin B |
| UBC | 9606.ENSP00000441543 | Ubiquitin C |
| UCHL1 | 9606.ENSP00000284440 | Ubiquitin C-terminal hydrolase L1 |
| USP9X | 9606.ENSP00000316357 | Ubiquitin specific peptidase 9, X-linked |
| VDAC1 | 9606.ENSP00000378487 | Voltage-dependent anion channel 1 |
| VPS35 | 9606.ENSP00000299138 | Vacuolar protein sorting 35 |
| ABL1 | 9606.ENSP00000361423 | ABL proto-oncogene 1 |
| BDNF | 9606.ENSP00000414303 | Brain-derived neurotrophic factor |
| CALM3 | 9606.ENSP00000291295 | Calmodulin 3 |
| CALML3 | 9606.ENSP00000315299 | Calmodulin-like 3 |
| CALML4 | 9606.ENSP00000419081 | Calmodulin-like 4 |
| CALML5 | 9606.ENSP00000369689 | Calmodulin-like 5 |
| CALML6 | 9606.ENSP00000304643 | Calmodulin-like 6 |
| CRYAB | 9606.ENSP00000433560 | Alpha-crystallin B chain |
| DNAJB1 | 9606.ENSP00000254322 | DnaJ heat shock protein family (Hsp40) member B1 |
| EGFR | 9606.ENSP00000275493 | Epidermal growth factor receptor |
| GAPDH | 9606.ENSP00000380070 | Glyceraldehyde-3-phosphate dehydrogenase |
| HDAC6 | 9606.ENSP00000365804 | Histone deacetylase 6 |
| HSP90AA1 | 9606.ENSP00000335153 | Heat shock protein 90 alpha family class A member 1 |
| HSPA1B | 9606.ENSP00000364801 | Heat shock protein family A, member 1B |
| HSPA4 | 9606.ENSP00000302961 | Heat shock protein family A, member 4 |
| HSPA5 | 9606.ENSP00000324173 | Heat shock protein family A, member 5 |
| HSPA8 | 9606.ENSP00000437125 | Heat shock protein family A, member 8 |
| HSPB1 | 9606.ENSP00000248553 | Heat shock protein beta-1 |
| HSPB2 | 9606.ENSP00000302476 | Heat shock protein beta-2 |
| IAPP | 9606.ENSP00000240652 | Islet amyloid polypeptide |
| INS | 9606.ENSP00000380432 | Insulin |
| NCL | 9606.ENSP00000318195 | Nucleolin |
| NRGN | 9606.ENSP00000284292 | Neurogranin |
| STUB1 | 9606.ENSP00000219548 | STIP1 homology and U-box containing protein 1 |
| TLR2 | 9606.ENSP00000260010 | Toll-like receptor 2 |
| TLR4 | 9606.ENSP00000363089 | Toll-like receptor 4 |
| SNAP25 | 9606.ENSP00000254976 | Synaptosome Associated Protein 25 |
| SYN1 | 9606.ENSP00000295987 | Synapsin I |
| SLC6A2 | 9606.ENSP00000219833 | Solute Carrier Family 6 Member 2 |
| SLC1A2 | 9606.ENSP00000278379 | Solute Carrier Family 1 Member 2 |
| NR4A2 | 9606.ENSP00000344479 | Nuclear Receptor Subfamily 4 Group A Member 2 |
| SNCB | 9606.ENSP00000377296 | Beta-Synuclein |
| RAB1A | 9606.ENSP00000387286 | Ras-related protein Rab-1A |
| SNCA | 9606.ENSP00000500990 | synuclein alpha |

Table S2. p-values and adjusted p-values of α-syn and its interactors for enrichment pathway analysis

| **Parameters** | **Term** | **P-value** | **Adjusted P-value** |
| --- | --- | --- | --- |
| **α-Syn** | Pathways of neurodegeneration | 3.53E^-29^ | 6.35E^-27^ |
|  | Parkinson disease | 1.33E^-27^ | 1.19E^-25^ |
|  | Lipid and atherosclerosis | 6.87E^-14^ | 4.12E^-12^ |
|  | Amphetamine addiction | 9.65E^-14^ | 4.34E^-12^ |
|  | Alcoholism | 5.41E^-12^ | 1.95E^-10^ |
|  | Dopaminergic synapse | 7.33E^-11^ | 2.20E^-09^ |
|  | Estrogen signaling pathway | 1.06E^-10^ | 2.73E^-09^ |
|  | Alzheimer disease | 1.24E^-09^ | 2.80E^-08^ |
|  | Amyotrophic lateral sclerosis | 1.20E^-08^ | 2.40E^-07^ |
|  | Neurotrophin signaling pathway | 1.67E^-08^ | 2.66E^-07^ |
|  |  |  |  |
| **α-Syn interactors** | Olfactory transduction | 9.92E^-36^ | 1.79E^-34^ |
|  | Porphyrin and chlorophyll metabolism | 0.01956379 | 0.155693772 |
|  | Malaria | 0.025948962 | 0.155693772 |
|  | Collecting duct acid secretion | 0.126656642 | 0.46758867 |
|  | Ascorbate and aldarate metabolism | 0.139709564 | 0.46758867 |
|  | Pentose and glucuronate interconversions | 0.15681347 | 0.46758867 |
|  | Glycine, serine and threonine metabolism | 0.181840038 | 0.46758867 |
|  | Steroid hormone biosynthesis | 0.263779141 | 0.538569303 |
|  | Retinol metabolism | 0.289243789 | 0.538569303 |
|  | Metabolism of xenobiotics by cytochrome P450 | 0.317279889 | 0.538569303 |

Table S3. Binding free-energy values (ΔG in kcal/mol) of bioactive compounds of the Lamiaceae family as target ligands along with α-Syn.

| **Major compounds** | **Binding free-energy values (ΔG in kcal/mol)** |
| --- | --- |
| 1,8-Cineole | -6.3 |
| 2-undecanone | -5.9 |
| 3-carene | -6.6 |
| Acetophenone | -5.9 |
| Apigenin | -8.9 |
| Aucubin | -8.2 |
| Betulinic acid | -7.7 |
| Bicyclogermacrene | -8.5 |
| Blumenol C | -7.5 |
| Borneol | -6.2 |
| Bornyl acetate | -6.9 |
| Caffeic acid | -6.9 |
| Camphene | -6.0 |
| Camphor | -6.5 |
| Carnosic acid | -7.7 |
| Carnosol | -9.4 |
| Carvacrol | -6.9 |
| Carvone | -6.8 |
| caryophyllene | -5.5 |
| caryophyllene oxide | -5.5 |
| cedryl acetate | -8.6 |
| Chlorogenic acid | -8.1 |
| Citronellol | -5.0 |
| eucalyptol | -6.3 |
| Eugenol | -6.4 |
| endo-Fenchol | -4.5 |
| Estragole | -5.1 |
| Fenchone | -6.7 |
| Geranial | -6.0 |
| Geraniol | -4.7 |
| Germacrene D | -8.8 |
| Guaiol | -6.3 |
| Harpagide | -8.0 |
| Hederagenin | -10.6 |
| Humulene | -5.7 |
| Kaempferol | -6.6 |
| Limonene | -6.7 |
| Linalool | -6.3 |
| Linalyl acetate | -7.0 |
| Linalool oxide | -6.4 |
| Luteolin | -9.3 |
| Menthol | -4.9 |
| Menthone | -6.5 |
| Menthyl acetate | -7.3 |
| Menthyl cinnamate | -6.6 |
| Methyl eugenol | -6.2 |
| Myricetin | -8.9 |
| Nerol acetate | -5.3 |
| Ocimene | -4.8 |
| Oleanolic acid | -10.9 |
| Patchouli alcohol | -7.0 |
| Perillaldehyde | -6.7 |
| Piperitenone | -5.0 |
| Piperitenone oxide | -5.1 |
| Pulegone | -7.1 |
| Quercetin | -9.7 |
| Quinolizidines | -5.8 |
| Rosmarinic acid | -7.3 |
| Sabinene | -4.2 |
| Scopoletin | -7.3 |
| spathulenol | -9.1 |
| Terpinen-4-ol | -6.8 |
| Terpinolene | -4.9 |
| thujanol | -6.4 |
| Thymol | -5.3 |
| Umbelliferone | -7.0 |
| Ursolic acid | -11.4 |
| α-campholenal | -6.2 |
| α-copaene | -8.6 |
| α-Pinene | -6.2 |
| α-Terpinene | -6.7 |
| α-Terpineol | -6.4 |
| α-terpinyl acetate | -7.7 |
| α-thujene | -6.1 |
| α-thujone | -6.7 |
| β-bourbonene | -6.0 |
| β-costol | -5.9 |
| β-Elemene | -7.8 |
| β-Eudesmol | -6.1 |
| β-Pinene | -6.3 |
| β-Sesquiphellandrene | -8.3 |
| γ-Eudesmol | -8.9 |
| γ-Terpinene | -6.8 |
| δ-Cadinol | -5.7 |
| ρ-cymene | -5.0 |
